# Electroacupuncture pretreatment attenuates cerebral ischemic injury through α7 nicotinic acetylcholine receptor-mediated inhibition of high-mobility group box 1 release in rats

**DOI:** 10.1186/1742-2094-9-24

**Published:** 2012-01-26

**Authors:** Qiang Wang, Feng Wang, Xin Li, Qianzi Yang, Xuying Li, Ning Xu, Yi Huang, Qiaomei Zhang, Xingchun Gou, Shaoyang Chen, Lize Xiong

**Affiliations:** 1 Department of Anesthesiology, Xijing Hospital, Fourth Military Medical University, Xi'an 710032, China

**Keywords:** α7 nicotinic acetylcholine receptor, Cerebral ischemia, Electroacupuncture, Pretreatment, High-mobility group box 1

## Abstract

**Background:**

We have previously reported that electroacupuncture (EA) pretreatment induced tolerance against cerebral ischemic injury, but the mechanisms underlying this effect of EA are unknown. In this study, we assessed the effect of EA pretreatment on the expression of α7 nicotinic acetylcholine receptors (α7nAChR), using the ischemia-reperfusion model of focal cerebral ischemia in rats. Further, we investigated the role of high mobility group box 1 (HMGB1) in neuroprotection mediated by the α7nAChR and EA.

**Methods:**

Rats were treated with EA at the acupoint "Baihui (GV 20)" 24 h before focal cerebral ischemia which was induced for 120 min by middle cerebral artery occlusion. Neurobehavioral scores, infarction volumes, neuronal apoptosis, and HMGB1 levels were evaluated after reperfusion. The α7nAChR agonist PHA-543613 and the antagonist α-bungarotoxin (α-BGT) were used to investigate the role of the α7nAChR in mediating neuroprotective effects. The roles of the α7nAChR and HMGB1 release in neuroprotection were further tested in neuronal cultures exposed to oxygen and glucose deprivation (OGD).

**Results:**

Our results showed that the expression of α7nAChR was significantly decreased after reperfusion. EA pretreatment prevented the reduction in neuronal expression of α7nAChR after reperfusion in the ischemic penumbra. Pretreatment with PHA-543613 afforded neuroprotective effects against ischemic damage. Moreover, EA pretreatment reduced infarct volume, improved neurological outcome, inhibited neuronal apoptosis and HMGB1 release following reperfusion, and the beneficial effects were attenuated by α-BGT. The HMGB1 levels in plasma and the penumbral brain tissue were correlated with the number of apoptotic neurons in the ischemic penumbra. Furthermore, OGD in cultured neurons triggered HMGB1 release into the culture medium, and this effect was efficiently suppressed by PHA-543,613. Pretreatment with α-BGT reversed the inhibitory effect of PHA-543,613 on HMGB1 release.

**Conclusion:**

These data demonstrate that EA pretreatment strongly protects the brain against transient cerebral ischemic injury, and inhibits HMGB1 release through α7nAChR activation in rats. These findings suggest the novel potential for stroke interventions harnessing the anti-inflammatory effects of α7nAChR activation, through acupuncture or pharmacological strategies.

## Background

One of the top killers of human, stroke claims hundreds of thousands of lives every year throughout the world. To fulfill the increasing need for effective and practical intervention strategies, it is important to gain a better understanding of neuroprotective mechanisms in the brain [1]. Preconditioning, as a potent endogenous protective maneuver, activates several endogenous signaling pathways that result in tolerance against ischemia. Identification of these pathways and their targets will likely contribute to the development of novel therapeutic concepts [2]. We previously reported that electroacupuncture (EA) pretreatment afforded strong protection against transient cerebral ischemic injury [3,4]. However, the signaling mechanisms mediating the effects of EA pretreatment are unclear.

Acute ischemic stroke involves a complex array of processes involving multiple biological systems, the combined action of which determines the outcome of the ischemic event [5]. Among these processes, a growing body of data implicates an integral role for inflammation, which forms an important component of the interactions between the nervous and immune systems in stroke pathology [6,7]. Endogenous anti-inflammatory mechanisms function to control the inflammatory response and prevent injury induced by an excessive immune response. The cholinergic anti-inflammatory pathway represents a physiological mechanism by which the nervous system interacts with the innate immune system to restrain systemic inflammatory responses [8]. There is convincing evidence of the critical importance of the α7 nicotinic acetylcholine receptor (α7nAChR) in mediating cholinergic anti-inflammatory signaling [9].

It is known that α7nAChR-dependent cholinergic signaling is implicated in suppressing the release of high mobility group box 1 (HMGB1) [10]. HMGB1 has important functions in mediating the pathology of acute damage and subsequent inflammatory processes in the post-ischemic brain [11-13]. Experimental and clinical studies have demonstrated that the anti-inflammatory actions of acupuncture are mediated via central inhibition of the innate immune system as a result of vagal activity [14]. Moreover, recent studies using animal models of Parkinson's disease and amyotrophic lateral sclerosis have shown that neuroprotection by acupuncture or EA may be mediated via inhibition of the neuroinflammatory response [15,16].

We hypothesized that EA may exert neuroprotective effects in stroke by regulating the expression of α7nAChR, and tested this using the middle cerebral artery occlusion (MCAO) model of focal ischemia in rats. We also investigated the possible role of HMGB1 suppression in mediating this effect, both in rats subjected to ischemia and in neuronal cultures subjected to oxygen- and glucose deprivation (OGD).

## Materials and methods

### Animal Care and Drugs

The experimental protocol used in this study was approved by the Ethics Committee for Animal Experimentation of the Fourth Military Medical University and was conducted according to the Guidelines for Animal Experimentation of the Fourth Military Medical University (Xi'an, China). Male Sprague-Dawley rats, weighing 280-320 g, were provided by the Experimental Animal Center of the Fourth Military Medical University, and housed under controlled conditions with 12-hour light/dark cycle, temperature of 21 ± 2°C, and 60-70% humidity, for at least one week prior to drug treatment or surgery. The rats were allowed for free access to standard rodent diet and tap water.

The specific α7nAChR antagonist α-bungarotoxin (α-BGT) was obtained from Alexis Biochemicals Corporation (San Diego, CA, USA), and was made up in 150 mM NaCl before use. Because α-BGT does not penetrate the blood-brain barrier, the drug was administered intracerebroventricularly at a dose of 0.5 μg/kg according to a previous study [17].

The N-[(3R)-1-Azabicyclo[2.2.2]oct-3-yl]furo[2,3-c]pyridine-5-carboxamide hydrochloride (PHA-543,613 hydrochloride) is a potent and selective agonist for the α7 subtype of neuronal nicotinic acetylcholine receptors, with a high level of brain penetration and good oral bioavailability [18]. PHA-543,613 hydrochloride was purchased from Tocris Bioscience (Ellisville, MO, USA), and was dissolved in saline before intraperitoneal injection (1.0 mg/kg) [18].

Dimethylsulfoxide (DMSO) and 3-(4,5-dimethylthiazol-2-yl)-2,5-diphenyltera-zolium bromide (MTT) were purchased from Sigma-Aldrich Co. (St. Louis, MO, USA). The lactate dehydrogenase (LDH) kit was provided by the Jiancheng Bioengineering Institute (Nanjing, China).

### Experimental protocols

The numbers of animals per group for various experiments at different time points are summarized in Table 1.

**Table 1:** Numbers of animals per group for various experiments at different time points

Protocol	Experiment	Time point	Group	Number	Total
Effect of EA pretreatment on α7nAChR expression	Western blot analysis Immunofluorescent staining	24 h, 48 h, 72 h 72 h	Sham, Control, EA	n = 5 n = 3	44
Neuroprotective effect of PHA-543,613 pretreatment	Neurological evaluation and TTC staining	72 h	MCAO, PHA-543,613+MCAO, Vehicle+MCAO	n = 10	30
Effect of α-BGT on EA pretreatment induced neuroprotection and HMGB1 release	Neurological evaluation and TTC staining	72 h	Sham, MCAO, EA+MCAO, α-BGT+EA, Vehicle+EA, α-BGT+MCAO	n = 10	120
	TUNEL staining	24 h		n = 5	
	ELISA for HMGB1 level	72 h		n = 5	

EA, electroacupuncture; HMGB1, high mobility group box 1; MCAO, middle cerebral artery occlusion; TTC, 2,3,5-triphenyltetrazolium chloride; TUNEL, terminal deoxynucleotidyl transferase-mediated dUDP-biotin nick end-labeling; α7nAChR, α7 nicotinic acetylcholine receptor; α-BGT, α-bungarotoxin.

To determine the effect of EA pretreatment on α7nAChR expression after ischemia-reperfusion, rats were randomly assigned to one of three groups: Sham, MCAO, or EA+MCAO (n = 8 rats/group). The rats in the Sham group underwent identical surgery without MCAO. The animals in the EA+MCAO group received daily EA preconditioning for consecutive 5 days, and were subjected to MCAO 24 h after the end of the last EA pretreatment. The expression of α7nAChR was determined by western blot analysis and immunofluorescent staining.

To evaluate the neuroprotective effect of pretreatment with PHA-543,613, rats were treated with 1 mg/kg PHA-543,613 or 1 mL vehicle (saline) once daily for 5 days, and were subjected to MCAO at 24 h after the end of the last PHA-543,613 pretreatment. The neurological scores and infarct volumes were evaluated at 72 h after reperfusion.

To assess the effect of α-BGT on EA-induced neuroprotection and HMGB1 release following cerebral ischemia-reperfusion, rats were randomly assigned to Sham, MCAO, EA+MCAO, α-BGT+MCAO, α-BGT+EA and vehicle+EA groups (n = 20 rats/group). The rats in the Sham, MCAO and EA+MCAO groups received the treatments described above. Rats in the α-BGT+MCAO group received intracerebroventricular α-BGT (0.5 μg/kg) once a day for 5 days and were subjected to MCAO at 24 h after the last injection. Rats in the vehicle+EA group and the α-BGT+EA group received saline (10 μL) and α-BGT (0.5 μg/kg), respectively, at 30 min before the onset of EA, once a day for 5 days, and were subjected to MCAO at 24 h after the end of the last EA pretreatment. The neurological scores, infarct volumes, neuronal apoptosis and HMGB1 levels in the penumbral brain tissue and plasma were evaluated at 72 h after reperfusion.

All animals were anesthetized with sodium pentobarbital (40 mg/kg, i.p.) while undergoing EA stimulation or surgery. During anesthesia, rats inhaled oxygen via a face mask at a flow rate of 1 L/min to avoid potential hypoxia associated with anesthesia.

To further investigate the involvement of inhibition of HMGB1 release through α7nAChR activation, neuronal cultures were exposed to OGD to simulate cerebral ischemia and the HMGB1 concentration in the culture medium was tested in the presence and absence of PHA-543,613.

### Intracerebral Ventricular Injection

The rats were anesthetized and placed in a stereotaxic apparatus (Narishige, Tokyo, Japan). A scalp incision was made and bregma was exposed. A burr hole was drilled in the bone 1.5 mm lateral and 1.0 mm posterior to bregma at the right hemisphere. A stainless steel 26-gauge cannula (Plastics One Inc., C315G) was slowly introduced through the burr hole into the right lateral ventricle (3.8 mm beneath the dural surface). The cannula was fixed by dental cement and four stainless steel screws secured to the skull and occluded. The rats were allowed to recover from surgery for at least 3 days before experiments began. Vehicle or α-BGT was infused into the right lateral ventricle using a 25 μL Hamilton syringe (80630, Hamilton Co., Reno, NV, USA) at a rate of 0.5 μL/min and the needle was left for an additional 5 min before withdrawal to prevent reflux. The 25 μL Hamilton syringe was connected to the cannula by a PE-50 catheter.

### Electroacupuncture Pretreatment

EA pretreatment was performed as previously described [3,4] at the acupoint "Baihui (GV 20)", which is located at the intersection of the sagittal midline and the line linking the two ears. Briefly, animals were anesthetized, and the acupoint "Baihui (GV 20)" was stimulated at an intensity of 1 mA and a frequency of 2/15 Hz for 30 min, using the Hwato Electronic Acupuncture Treatment Instrument (Model No. SDZ-V, Suzhou Medical Appliances Co., Ltd., Suzhou, China). The core temperature of all the rats was maintained at 37.0 ± 0.5°C during EA pretreatment by surface heating or cooling (Spacelabs Medical Inc., Redmond, WA). The right femoral artery was cannulated for continuous monitoring of blood pressure and for arterial blood sampling. Arterial blood gases and plasma glucose were measured at the onset of EA, 15 min after EA and at the end of EA. pO_2_, pCO_2_, and pH were quantified using a blood gas analyzer (ABL700, Radiometer, Denmark), and plasma glucose was measured with a blood glucose meter (OneTouch UltraEasy, Johnson & Johnson, USA).

### Transient Focal Cerebral Ischemia

Focal cerebral ischemia was induced by MCAO using an intraluminal filament technique as described in our previous studies [4,19]. Rats were placed in a stereotaxic frame, and a scalpel was used to make an incision on the scalp and carefully scrape the exposed skull. The skull (4 mm lateral and 2 mm caudal to bregma) was thinned without perforation by a drill. Regional cerebral blood flow (rCBF) was monitored through a disposable microtip fiber optic probe (diameter 0.5 mm), which was attached to the skull with a probe holder, and connected through a Master Probe to a laser Doppler computerized main unit (PeriFlux 5000, Perimed AB, Sweden). The MCAO was considered adequate if the rCBF showed a sharp drop to 20% and recovered up to more than 80% of baseline (pre-ischemia) level; animals that did not meet this requirement were excluded.

### Neurobehavioral Evaluation and Infarct Assessment

At 72 h after reperfusion, the animals were neurologically assessed by an investigator who was unaware to animal grouping. An 18-point scoring system reported by Garcia *et al*.[20] with modifications was used for neurologic assessment.

The infarct volume was assessed by 2,3,5-triphenyltetrazolium chloride (TTC, Sigma-Aldrich, St. Louis, MO) staining as previously described [4,19]. At 72 h after reperfusion, the rats were re-anesthetized with 4% isoflurane in oxygen and decapitated. The brains were rapidly removed and were sliced into 2-mm thick coronal sections with the aid of a brain matrix. Sections were stained with standard 2% TTC, for 10 minutes at 37°C, followed by overnight immersion in 4% paraformaldehyde, and were photographed with a digital camera (Kodak DC240, Eastman Kodak Co., Rochester) connected to a computer. Unstained areas were defined as infarct, and were measured using Adobe Photoshop 8.0 CS for Windows by an investigator blinded to the experimental grouping. To account for cerebral edema and differential shrinkage resulting from tissue processing, the total volume of the infarction was presented as a percentage of the contralateral hemisphere.

### TUNEL Staining

Samples from 5 groups (MCAO, EA+MCAO, α-BGT+EA, vehicle+EA, α-BGT+MCAO, n = 5 per group) in α7nAChR-blocking experiments were used for TUNEL staining. Twenty-four hours after reperfusion, neuronal apoptosis in the ischemic penumbra was assessed *in situ *by TUNEL staining as described in our previous study [4]. The TUNEL staining was quantitatively evaluated using the method described by Wang *et al. *[21]. Briefly, 32 pixels of 0.10 mm^2 ^were located under a light microscope with 100 × magnification, and the total number of positively stained cells in these pixels was counted and expressed as cells/mm^2^.

### Western Blot Analysis of α7nAChR

At 24 h, 48 h, and 72 h after reperfusion, rats (n = 5 per time point per group) were deeply anesthetized, and the ischemic penumbras were microdissected according to established protocols in rodent models of unilateral proximal MCAO [22,23]. Briefly, a 4-mm thick coronal brain slice was cut, beginning 6 mm from the anterior tip of the frontal lobe. Then a longitudinal cut (from top to bottom) was made approximately 2 mm from the midline through the ischemic hemisphere to remove medial parts. Finally, a transverse diagonal cut was made at approximately the "2 o'clock" position to separate the wedge-shaped penumbra. The tissue was homogenized on ice in RIPA lysis buffer (Beyotime, Nantong, China) with 1 × Roche complete protease inhibitor cocktail and 1 mM phenylmethylsulfonyl fluoride (PMSF), and western blot was performed as previously described [10]. The following primary antibodies were used in this study: anti-α7nAChR rabbit polyclonal antibody (Santa Cruz Biotechnology, Cat# sc-5544, 1:200 dilution) and anti-β-actin mouse monoclonal antibody (Santa Cruz Biotechnology, Cat# sc-130300, 1:1000 dilution). Appropriate secondary horseradish peroxidase-conjugated goat anti-rabbit (Cat# 32460) or goat anti-mouse antibodies (Cat# 32430) (Pierce Biotechnology Inc., 1:5000 dilution) were used.

Semi-quantitative analysis of the blots was performed using densitometry followed by quantification with the NIH image program (NIH Image Version 1.61). Each sample was subjected to immunoblotting three times, and the final optical density value (relative to that for the internal standard) represents the average of these three separate analyses.

### Double Immunofluorescence for α7nAChR and NeuN

At 72 h after reperfusion, the rats (n = 3 for each group) were deeply anesthetized and transcardially perfused with PBS and 4% paraformaldehyde as described previously [4]. To ensure that homologous areas of injury were sampled between animals, parallel sets of sections from -3.0 to -5.0 mm from Bregma (covering the infarct area) were used. The 10-μm thick coronal sections were incubated for 12 h at room temperature with the following primary antibodies: anti-α7nAChR rabbit polyclonal antibody (Santa Cruz Biotechnology, Cat# sc-5544, 1:200 dilution) and anti-NeuN mouse monoclonal antibody (Chemicon International Inc., Cat# MAB377, 1:1000 dilution). After washing three times with PBS, sections were incubated for 1 h at room temperature with FITC-labeled goat-anti mouse IgG (Molecular Probes Inc., Cat# 62-6511, 1:800 dilution) and Alexa Fluor 594-labeled goat-anti rabbit IgG (Invitrogen, Cat# A11012, 1:800 dilution). Sections incubated without primary or secondary antibodies served as negative controls. Finally, sections were observed and images were captured using an Olympus BX-60 fluorescence microscope (Olympus Corporation, Japan).

### Neuronal cultures and OGD

Neuronal cultures were prepared from 17-19 day embryos of Sprague-Dawley rats. The cortical neurons were suspended in neuron-defined culture medium and plated onto poly-L-lysine-coated 35-mm dishes (3 × 10^5 ^cells per dish). Neurobasal medium supplemented with 2% B27, 0.3 mM L-glutamine and 1% penicillin-streptomycin was used (Invitrogen Gibco, NY, USA). Half of the medium was replaced every 3 days. The purity of the neuronal cultures was confirmed by microtubule associated proteins-2 (MAP-2) staining showing that the cultures were > 95% neurons.

After 13 days *in vitro*, neurons were exposed to OGD as previously described [24]. In brief, the culture medium was replaced with serum- and glucose-free medium and placed in an incubator chamber (Billups-Rothenburg, CA, USA), which was flushed for 5 min with 95% nitrogen and 5% CO_2 _and then sealed at 37°C for 90 min (hypoxia). Control cultures were incubated for the same period of time at 37°C in a humidified atmosphere of 95% air and 5% CO_2_.

The PHA-543,613 (0.1, 0.5, 2.5, and 12.5 μM) was added to neuronal cultures immediately preceding OGD. In separate α7nAChR-blocking experiments, we pre-treated neuronal cultures with α-BGT (10 nM) for 30 min at 37°C prior to OGD in the presence or absence of PHA-543,613. The concentration of α-BGT in this experiment was chosen based on previous studies [25,26], in which 10 nM of α-BGT did not show cytotoxicity, but could essentially block α7nAChR-mediated biological effects.

### MTT cell survival assay and Lactate dehydrogenase release

At 24 h after the exposure of the neuronal monocultures to OGD, cell viability was assessed by the ability of cells to take up MTT [24]. In brief, aliquots of MTT solution (final concentration of 0.2 mg/mL) were added to medium and the cells were incubated for 4 h at 37°C. After the medium had been removed, the dye crystals were solubilized by adding 200 μl of DMSO, and the absorption at 570 nm was measured with a Model 550 microplate reader (Bio-Rad lab, California, USA). The relative cell viability for each group was expressed as a percentage of the mean OD_570 _value of the control group.

Lactate dehydrogenase (LDH) release is an indicator of plasma membrane damage and is commonly used for the assessment of necrotic cell death. At 24 h after OGD, LDH activity in the culture medium was determined using a commercially available assay (Jiancheng Bioengineering Institute, Nanjing, China) according to the manufacturer's protocol. The activity unit was defined as units per deciliter.

### HMGB1 analysis

At 72 h after reperfusion, blood samples (1 mL, n = 5 per group) were collected via cardiac puncture immediately following euthanasia, then centrifuged at 2,000 g for 20 min at 4°C. After centrifugation, the plasma was frozen at -80°C until further testing. Samples of ischemic penumbra were microdissected and weighed. The cytoplasmic fraction of brain tissue was prepared as previously reported [27]. In brief, the brain tissues of the ischemic penumbra were gently homogenized in 10 mM N-2-hydroxyethylpiperazine-N'-ethanesulfonic acid (HEPES)/10 mM KCl buffer with 0.08% NP-40, 0.1 mM ethylenediaminetetraacetic acid (EDTA), 0.5 mM dithiothreitol (DTT), and 0.5 mM phenylmethylsulfonyl fluoride (PMSF), and the soluble fraction derived from the cytoplasm was kept at -80°C until further testing.

The HMGB1 levels in the neuronal culture medium, the brain cytoplasmic fraction, and the plasma were analyzed with an HMGB1 ELISA Kit II (Shino-test Corporation, Kanagawa, Japan). The cytoplasmic protein concentration was determined as described by Bradford [28]. The final results were presented as ng/mg protein in cytoplasm and as ng/mL in culture medium and plasma.

### Statistical Analysis

Brain sections were examined by two independent and blinded investigators. SPSS 13.0 for Windows (SPSS Inc., Chicago, IL) was used to conduct statistical analyses. All values, except for neurological scores, were presented as mean ± SEM, and were analyzed by one-way analysis of variance. Between-group differences were detected with Tukey's *post hoc *tests. The neurological deficit scores were expressed as median (interquartile range) and were analyzed with Kruskal-Wallis test followed by the Mann-Whitney *U *test with Bonferroni correction. The correlation between the numbers of apoptotic neuronal cells in the ischemic penumbra and HMGB1 levels was analyzed statistically using Pearson's correlation coefficient. A P value of less than 0.05 was considered to be statistically significant.

## Results

### Physiological Parameters

There were no significant differences in physiological parameters during EA pretreatment (at the onset of EA, 15 min after EA and the end of EA) or surgery (at the onset of ischemia, 60 min after the onset of ischemia, and 30 min after reperfusion). Arterial blood gases (pO_2_, pCO_2_, pH), mean arterial pressure, body temperature and plasma glucose remained in the normal range during the experimental period. Regional cerebral blood flow (rCBF) was monitored in the period of transient focal cerebral ischemia and reperfusion in order to meet the ischemic requirement (data not shown).

### Expression of α7nAChR following ischemia-reperfusion

**Figure 1:**
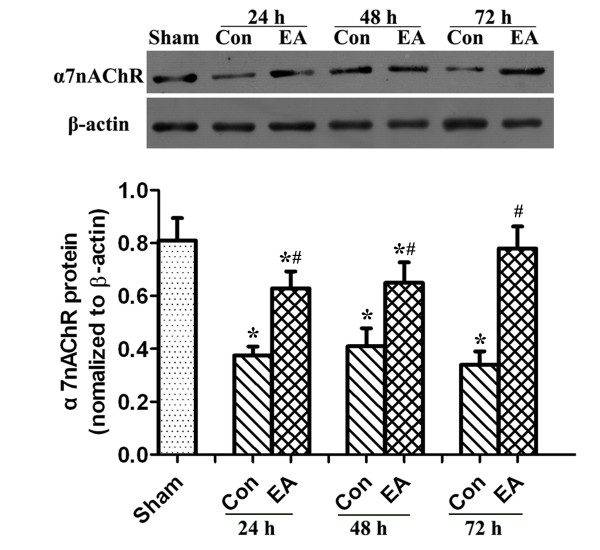
**Temporal expression of α7nAChR protein in the ischemic penumbra**. **(A) **Western blot showing representative α7nAChR protein expression at 24 h, 48 h, and 72 h after reperfusion, in the ischemic penumbra and in the sham-operated group. **(B) **The density of α7nAChR protein expression relative to β-actin protein expression. Data are means ± SEM (n = 5 per time point per group). *P < 0.01 *vs*. Sham, #P < 0.05 *vs*. Con.

The expression of α7nAChR was significantly decreased in the MCAO group compared to Sham animals (P < 0.01). EA pretreatment produced an evident inhibition of this reduction at 24 h, 48 h and 72 h after reperfusion (P < 0.05). There was no difference in α7nAChR expression between different time points within groups (Figure 1).

### Neuronal expression of α7nAChR in ischemic penumbra

**Figure 2:**
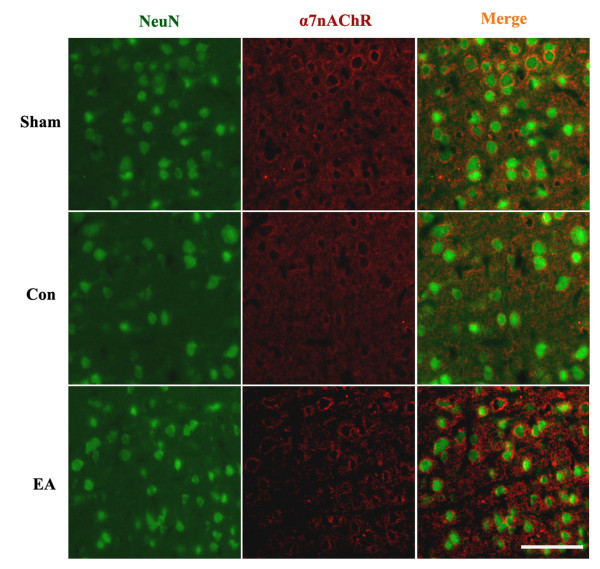
**Immunofluorescent double-labeling of α7nAChR and NeuN**. Representative double immunofluorescence staining for NeuN (neuronal marker, green) and α7nAChR (red) in the ischemic penumbra (n = 3). The α7nAChR was colocalized with neurons at 72 h after reperfusion. EA pretreatment suppressed the down-regulation of neuronal α7nAChR in the ischemic penumbra following reperfusion. Scale bars = 50 μm.

As shown in Figure 2, α7nAChR expression was observed throughout the hemisphere in the Sham group, whereas it was significantly decreased in the ischemic penumbra in the MCAO group. EA pretreatment inhibited this reduction at 24 h, 48 h and 72 h after reperfusion. Double immunofluorescence for α7nAChR and NeuN showed that α7nAChR was mainly localized in neurons.

### PHA 543613 Pretreatment Induces Neuroprotection

**Figure 3:**
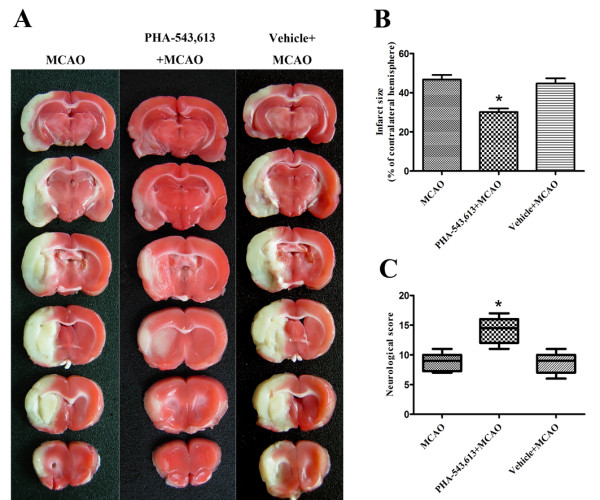
**Neuroprotection by pretreatment with PHA-543,613**. **(A) **Representative photographs of coronal brain sections following infarction, stained with 2,3,5-triphenyltetrazolium chloride. Red area is healthy tissue; white area is infarct tissue. Pretreatment with PHA-543,613 significantly reduced the infarction volumes **(B) **and improved the neurological scores **(C) **compared with the MCAO group. (n = 10) *P < 0.05 *vs*. MCAO and vehicle groups.

Pretreatment with the α7nAChR agonist PHA-543,613 significantly improved the neurological scores and reduced the infarction volumes measured at 72 h after reperfusion compared with the MCAO group (P < 0.05). There was no statistically significant difference between the MCAO group and the vehicle+MCAO group (Figure 3).

### Effect of α-BGT on neuroprotection induced by EA pretreatment

**Figure 4:**
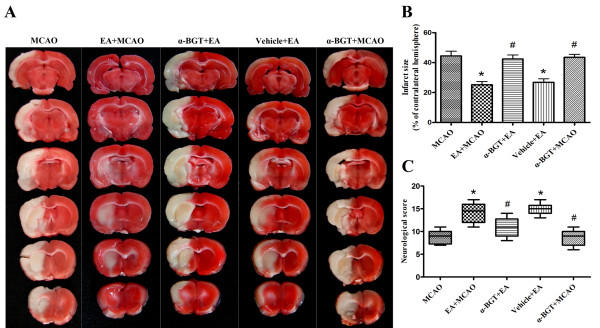
**Neurological scores and infarct sizes at 72 h after reperfusion**. **(A) **Representative photographs of coronal brain sections following infarction, stained with 2,3,5-triphenyltetrazolium chloride. Red area is healthy tissue; white area is infarct tissue. Pretreatment with EA significantly reduced infarct sizes **(B) **and improved neurological scores **(C) **and the α7nAChR antagonist α-BGT reversed the beneficial effect of EA pretreatment (n = 10). *P < 0.01 *vs*. MCAO, #P < 0.01 *vs*. EA+MCAO.

The effects of α-BGT on neuroprotection induced by EA pretreatment are summarized in Figure 4. At 72 h after reperfusion, the EA+MCAO group showed smaller brain infarct volumes compared with the MCAO group (P < 0.01). In the α-BGT+EA group, the infarct volume was similar to that in the MCAO group, and larger than that in the EA+MCAO group (P < 0.01). The average infarct volume in the vehicle+EA group was significantly different from that in the MCAO group (P < 0.01), and was similar to that in the EA+MCAO group. The infarct volume in the α-BGT+MCAO group was not significantly different from that in the MCAO group (Figure 4B). A similar blocking effect of α-BGT on the effect of EA was observed for the neurological scores (Figure 4C).

### Effect of α-BGT on neuronal apoptosis in ischemic penumbra

**Figure 5:**
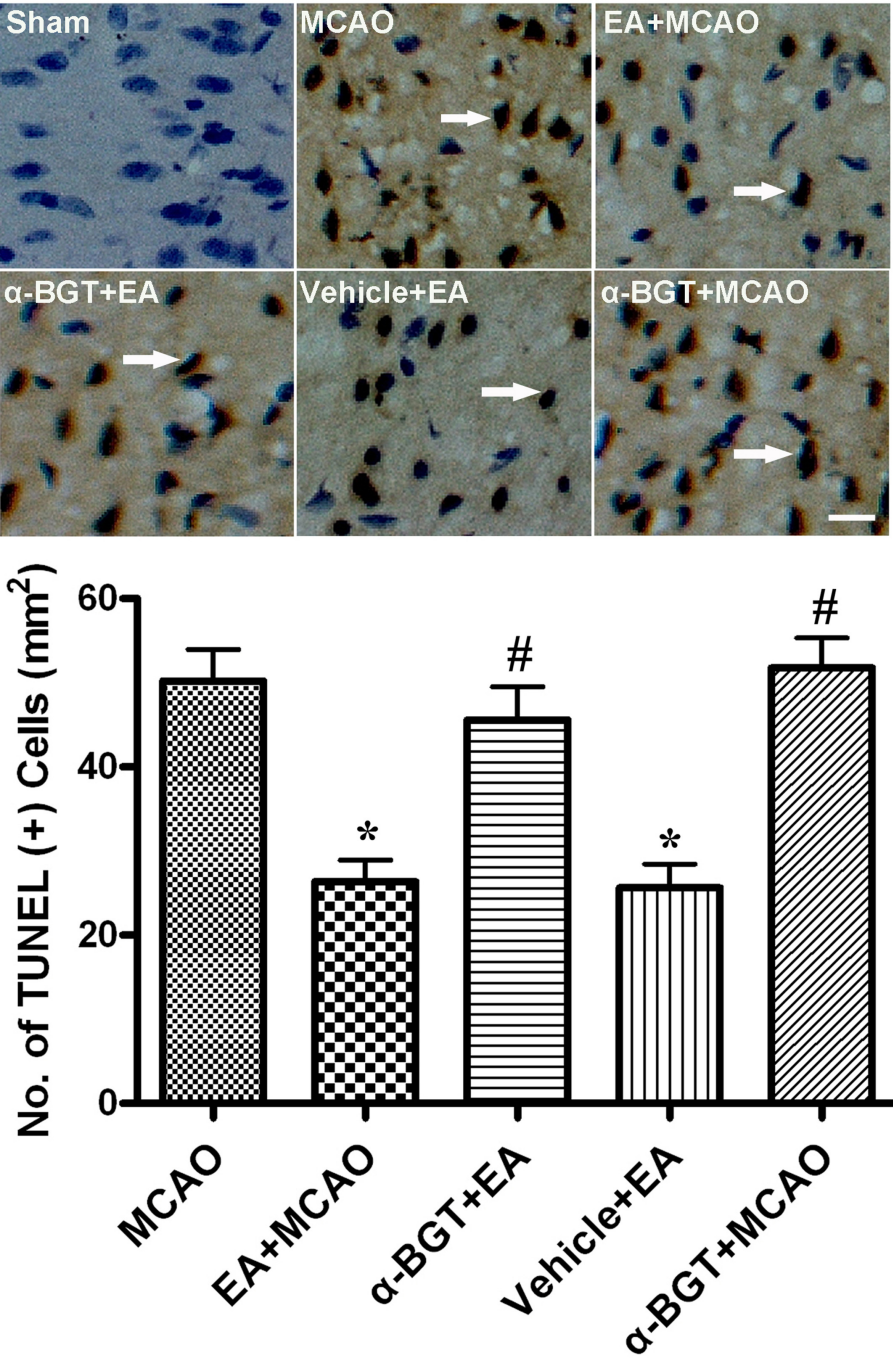
**Neuronal apoptosis at 72 h after reperfusion**. (***Upper***) Representative photomicrographs of TUNEL staining in the ischemic penumbra. The arrowhead indicates TUNEL-positive cell. Scale bars = 20 μm. (***Bottom***) Quantitative analysis of the number of TUNEL-positive cells in the ischemic penumbra. Pretreatment with EA significantly decreased the number of TUNEL-positive cells, while the α7nAChR antagonist α-BGT attenuated the reduction (n = 5). *P < 0.01 *vs*. MCAO, #P < 0.01 *vs*. EA+MCAO.

As shown in Figure 5, little positive TUNEL staining (brown) was detected in brain sections of sham animals at 72 h after reperfusion, whereas large numbers of TUNEL-positive cells were seen in the ischemic penumbra in the MCAO, α-BGT+EA and α-BGT+MCAO groups. In contrast, only a small amount of TUNEL staining was observed in the EA+MCAO and vehicle+EA groups. Quantitative analysis showed that the EA pretreatment significantly reduced the number of TUNEL-positive cells, compared to the MCAO, α-BGT+EA and α-BGT+MCAO groups. There was no difference between the MCAO, α-BGT+EA and α-BGT+MCAO groups.

### HMGB1 levels in the penumbral brain tissue and plasma

**Figure 6:**
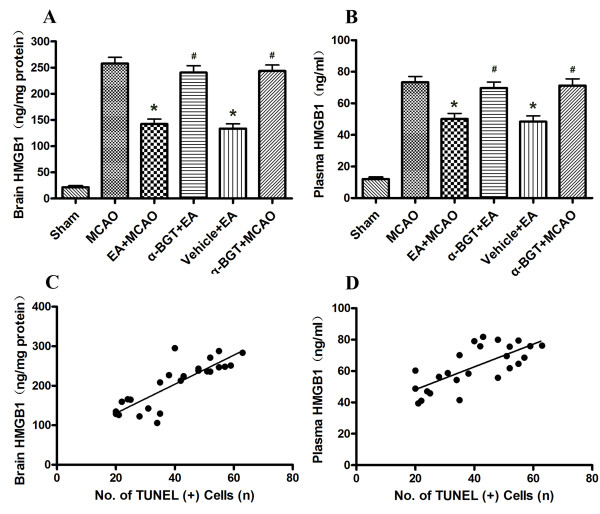
**Levels of high-mobility group box 1 (HMGB1) in the penumbral cytoplasm and plasma**. **(A) **HMGB1 content in the penumbral cytoplasm 72 h after reperfusion. **(B) **HMGB1 concentration in plasma 72 h after reperfusion. Each value represents the mean ± SEM of five measurements. *P < 0.01 *vs*. MCAO, #P < 0.01 *vs*. EA+MCAO. **(C) **The relationship between the numbers of apoptotic neuronal cells and brain HMGB1 content (n = 25 pairs, *r *= 0.8409, P < 0.01). **(D) **The relationship between the numbers of apoptotic neuronal cells and plasma HMGB1 concentrations (n = 25 pairs, *r *= 0.7263, P < 0.01).

Figure 6 shows the levels of HMGB1 in the penumbral brain tissue and plasma at 72 h after reperfusion. The HMGB1 content in the penumbra was significantly increased in the MCAO group compared to that in the Sham group (P < 0.01). The HMGB1 content was significantly lower in the EA+MCAO and vehicle+EA groups compared to that in the MCAO group (P < 0.01). There was no difference between the MCAO, α-BGT+EA and α-BGT+MCAO groups (Figure 6A). Similar changes were observed for plasma HMGB1 levels (Figure 6B).

We found significant positive correlations between the numbers of apoptotic neurons and the brain HMGB1 content (*r *= 0.8409, P < 0.01; Figure 6C) and the number of apoptotic neurons and the plasma HMGB1 concentration (*r *= 0.7263, P < 0.01; Figure 6D).

### Involvement of α7nAChR in OGD-induced HMGB1 release in primary neurons

**Figure 7:**
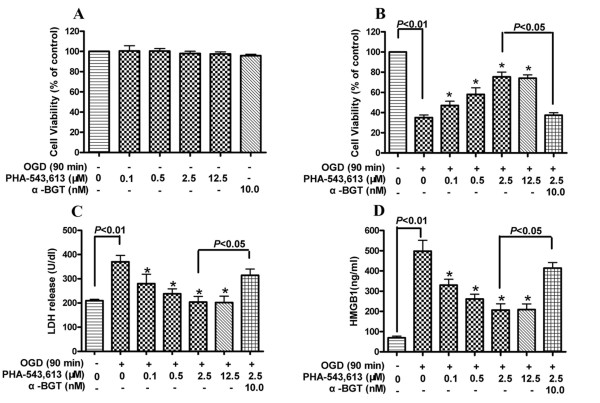
**Involvement of α7nAChR in OGD-induced HMGB1 release in primary cultured neurons**. **(A) **Effect of PHA-543,613 and α-BGT on cell viability in normal cultured primary neurons. **(B-D) **Effects of PHA-543,613 and α-BGT in neurons exposed to OGD on cell viability (**B**), LDH release (**C**) and HMGB1 levels (**D**). The PHA-543,613 (0-12.5 μM) and α-BGT (10 nM) were incubated with primary cultured neurons for 24 h. PHA-543,613 was added immediately before the onset of OGD. The α-BGT was pre-incubated with neurons for 30 min before the onset of OGD. The control cells (0 μM) received vehicle (saline). The cell viability (MTT assay), LDH release and HMGB1 level were tested at 24 h after the exposure of cell cultures to OGD. *P < 0.05 compared with the 0 μM group exposed to OGD.

To ensure that HMGB1 release was not caused by non-specific protein release due to cell damage, the toxicity of PHA-543,613 (0.1-12.5 μM) and α-BGT (10 nM) was tested under conditions of normoxia using the MTT assay. The drugs did not have any influence on cell viability at these concentrations after incubation for 24 h (Figure 7A).

The viability of neurons was assessed at 24 h after a 90-min exposure to OGD. PHA-543,613 increased the viability of neurons exposed to OGD in a concentration-dependent manner (Figure 7B) and attenuated LDH release resulting from OGD (Figure 7C). However, pretreatment with α-BGT completely abolished the protective effect of PHA-543,613. Exposure of neurons to OGD for 90 min triggered HMGB1 release into the culture medium, and this was efficiently suppressed by PHA-543,613 in a concentration-dependent manner. Pretreatment with α-BGT reversed the inhibitory effect of PHA-543,613 on HMGB1 release (Figure 7D).

## Discussion

Focal cerebral ischemia evokes a robust inflammatory response that begins within a few hours of onset and typifies the secondary or delayed response to ischemia. Activation of the innate immune response is a consequence of stroke, and preclinical data indicate that inflammation in the immediate post-stroke period contributes to ischemic brain injury [29]. Nervous system interaction with the immune system is vital for modulating innate immune responses and controlling inflammation [6]. The cholinergic anti-inflammatory pathway is a neural α7AChR-dependent mechanism that suppresses the innate inflammatory response [9,30], and provides novel opportunity for improving stroke therapy. The neuroprotective effects of α7AChR in the pathophysiology of cerebral ischemia-reperfusion have recently been reported by several investigators [31,32]. However, the expression and role of α7AChR in neuroprotection by EA pretreatment is still unclear.

In the present study, we found that α7AChR protein expression began to decrease in the ischemic penumbra at 24 h and remained low for at least 72 h following reperfusion. EA pretreatment reversed this reduction in α7AChR expression. In addition, our immunofluorescent staining showed that the α7AChR immunoreactivities were colocalized with NeuN immunoreactivities, indicating that the effect of EA pretreatment on α7nAChR expression was neuron-specific. These results are consistent with the possibility that α7AChR downregulation plays a role in producing neuronal damage in ischemia, and that EA may be neuroprotective by preventing this mechanism.

Activation of α7AChR with PHA-543,613 pretreatment significantly improved the neurological scores and reduced the infarction volumes. Moreover, the α7AChR antagonist α-BGT attenuated the beneficial effects of EA on infarct volumes, neurological outcomes, and apoptosis. Given alone, α-BGT had no significant effect on neurological outcomes, infarct volume, or neuronal apoptosis, indicating that α7AChRs blockade did not have any direct aggravating effect in these experiments. These results suggest that activation of the α7nAChR may be an essential mediator of the EA effect, acting to switch the cells from a vulnerable to a tolerant state.

There is a wealth of evidence to support that the systemic inflammatory response associated with ischemia-reperfusion injury contributes to the morbidity and mortality associated with stroke [7,29]. HMGB1, originally identified as a nuclear DNA-binding protein, has recently been characterized as a cytokine-like mediator of systemic inflammation [10]. In the pathophysiology of cerebral ischemia-reperfusion, HMGB1 is released from neurons early following ischemic injury, and acts as a mediator linking acute brain damage and subsequent inflammatory processes [11-13]. Our finding of increased HMGB1 levels in the brain cytoplasm and plasma suggest that exacerbated HMGB1 release may be triggered by ischemia/reperfusion and play a role in enhancing the inflammatory response. The elevation of plasma HMGB1 may be associated with the passive release of HMGB1 from the damaged brain. Consistent with this, serum HMGB1 levels were significantly elevated in patients with cerebral ischemia [33], suggesting the potential role of this protein as a therapeutic target. Intravenous injection of neutralizing anti-HMGB1 monoclonal antibody has been suggested as a novel therapeutic strategy for ischemic stroke [34]. We found that EA pretreatment counteracted the increase in HMGB1 levels in brain and plasma after reperfusion, and these beneficial effects were attenuated by α7nAChR blockade. Thus, EA pretreatment may suppress HMGB1 after ischemia-reperfusion via the activation of α7nAChR.

The findings that α7nAChR activation protected against OGD-induced cellular damage in cultured neurons, and that this action was blocked by an α7nAChR antagonist, confirms the neuroprotective role of this receptor. The increased HMGB1 released into the culture medium seen after OGD is consistent with our *in vivo *data, and the suppression of HMGB1 release by the α7nAChR agonist argues for an important role of the cholinergic system in protecting against this effect. This was further supported by the abolishment of the α7nAChR-mediated protection by pretreatment with the α7nAChR antagonist α-BGT. Taken together, these findings are consistent with a protective effect of α7nAChR-mediated inhibition of HMGB1 release after cerebral ischemia, and indicate that EA pretreatment can activate this protective pathway.

There is compelling evidence that neurons in the ischemic penumbra may undergo apoptosis after several hours or days following transient cerebral ischemia, suggesting that there is a time window following the onset of stroke in which recovery is possible. Apoptosis is known to contribute to cell death of ischemic brain tissue, and inhibition of apoptosis reduces ischemic injury [35]. We found numerous apoptotic neurons in the ischemic penumbra of controlled ischemic animals, whereas EA pretreatment significantly reduced the number of apoptotic neurons, suggesting that EA pretreatment alleviates neuronal apoptosis. Moreover, HMGB1 levels in the penumbral brain tissue and plasma correlated well with the numbers of apoptotic neuronal cells in the ischemic penumbra, potentially suggesting a role of HMGB1 inhibition in the alleviation of neuronal apoptosis by EA. In accordance with our experimental results, other *in vitro *studies have shown that HMGB1 release from macrophages was correlated with the occurrence of apoptosis, and it was suggested that these processes reflect common mechanisms [36]. However, other studies have shown that HMGB1 production occurred downstream of apoptosis in the final common pathway to organ damage in severe sepsis [37]. Thus, the crosstalk between HMGB1 and apoptosis needs to be further explored.

## Conclusion

In summary, our results strongly suggest that EA pretreatment affords strong protection against transient cerebral ischemic injury, and inhibits HMGB1 release through α7nAChR activation in rats. Although further studies are needed to elucidate the exact signaling cascades between α7nAChR and HMGB1, and to understand the crosstalk between HMGB1 and apoptosis, our findings may represent a novel mechanism of EA-induced tolerance to focal cerebral ischemia in rats. Further, the results also provide strong support for the possibility of α7nAChR-mediated anti-inflammatory interventions after stroke.

## Abbreviations

**DMSO**: dimethylsulfoxide; **EA**: electroacupuncture; **HMGB1**: high mobility group box 1; LDH: lactate dehydrogenase; **MCAO**: middle cerebral artery occlusion; **MTT**: 3-(4,5-dimethylthiazol-2-yl)-2,5-diphenyltera-zolium bromide; **OGD**: oxygen and glucose deprivation; **rCBF**: regional cerebral blood flow; **TTC**: 2,3,5-triphenyltetrazolium chloride; **TUNEL**, terminal deoxynucleotidyl transferase-mediated dUDP-biotin nick end-labeling; **α7nAChR**: α7 nicotinic acetylcholine receptor; **α-BGT**: α-bungarotoxin.

## Competing interests

The authors declare that they have no competing interests.

## Authors' contributions

QW handled funding and supervision, analyzed and interpreted the data, performed statistical analysis and drafted the manuscript. FW acquired the data, analyzed and interpreted the data and drafted the manuscript. XL acquired the data, analyzed and interpreted the data and performed statistical analysis. QY, XL, NX, YH, QZ and XG acquired the data. SC analyzed and interpreted the data and made critical revision of the manuscript for important intellectual content. LX conceived and designed the study, handled funding and supervision and made critical revision of the manuscript for important intellectual content. All authors read and approved the final manuscript.
